# Cluster of differentiation 38 monoclonal antibody therapy in the treatment of multiple myeloma: a systematic review and meta-analysis

**DOI:** 10.3389/fphar.2025.1687718

**Published:** 2026-02-12

**Authors:** Yan-Jun Guo, Zhi-Zhong Tian, Xiao-Fen Zhang, Zhen-Jun Zhu

**Affiliations:** 1 Department of Orthopedics, Xinxiang Central Hospital, The Fourth Clinical College of Xinxiang Medical University, Xinxiang, Henan, China; 2 Department of Hematology, The Second Affiliated Hospital of Shandong First Medical University, Tai’an, Shandong, China

**Keywords:** CD38 monoclonal antibody, cluster of differentiation 38, daratumumab, isatuximab, meta-analysis, multiple myeloma

## Abstract

**Background:**

Cluster of differentiation 38 (CD38) monoclonal antibodies, including daratumumab and isatuximab, have demonstrated clinical activity in relapsed or refractory multiple myeloma (MM). This study aims to systematically evaluate the efficacy and safety of CD38-targeted monoclonal antibodies compared with those of standard regimens.

**Methods:**

This study searched PubMed, the Cochrane Library, Web of Science, and Embase from inception until 30 June 2025 for randomized controlled trials (RCTs) comparing CD38 antibodies (alone or in combination) with proteasome inhibitor- or immunomodulatory agent-based control regimens in adults with multiple myeloma. Two reviewers independently screened the studies, extracted data, and assessed the risk of bias (Cochrane risk of bias 2.0). Pooled risk ratios (RRs) and hazard ratios (HRs) were estimated using fixed- or random-effects models according to I^2^ heterogeneity. Publication bias was examined using Egger’s test.

**Results:**

A total of eight RCTs with 2,821 patients were included. The pooled overall response rate (ORR) was significantly improved with CD38-targeted therapies (RR 1.59; 95% confidence interval [CI] 1.32–1.92; *p* < 0.001). Progression‐free survival (PFS) was also significantly prolonged (HR 0.50; 95% CI 0.39–0.61; *p* < 0.001). Subgroup analyses indicated consistent benefits across the renal function, age groups, and prior therapy lines. However, CD38-targeted therapies were associated with higher rates of non-hematologic adverse events, including infections and diarrhea.

**Conclusion:**

CD38 monoclonal antibodies enhance the depth of response and prolong progression-free survival in multiple myeloma, with an acceptable safety profile, supporting their integration into treatment algorithms.

## 1 Introduction

Multiple myeloma (MM) is a clonal plasma cell malignancy characterized by aberrant proliferation within the bone marrow microenvironment, production of monoclonal immunoglobulin, and associated end-organ damage including osteolytic lesions, renal impairment, and bone marrow failure (Hu et al., 2024; Taranto et al., 2024). Despite advances in proteasome inhibitors, immunomodulatory agents, and autologous stem-cell transplantation, MM remains incurable, with the median overall survival (OS) ranging from 3 to 5 years in newly diagnosed patients and substantially lower in the relapsed or refractory setting (Zhang et al., 2024; Jiang and Zhou, 2024). Resistance to established therapies and cumulative toxicity underscore the urgent need for novel, targeted approaches that achieve deeper and more durable responses (Wu and Zhao, 2022; Gozzetti et al., 2022; Bisht et al., 2023).

Cluster of differentiation 38 (CD38) is a type-II transmembrane glycoprotein that is highly expressed on plasma cells and recognized as a multifunctional ectoenzyme involved in calcium signaling, cell adhesion, and immune modulation (Liu et al., 2024). Its uniform and high-level expression on malignant plasma cells, contrasted with the more restricted expression on normal hematopoietic precursors, renders CD38 a favored immunotherapeutic target. Monoclonal antibodies directed against CD38 mediate antitumor activity through multiple mechanisms, including complement-dependent cytotoxicity, antibody-dependent cellular cytotoxicity, antibody-dependent cellular phagocytosis, and direct induction of apoptosis. In addition, CD38-targeted antibodies may disrupt the immunosuppressive tumor microenvironment by depleting regulatory immune subsets and modulating adenosine metabolism (van de Donk and Usmani, 2018; Lin et al., 2024). Daratumumab, the first anti-CD38 monoclonal antibody approved for MM, demonstrated significant efficacy both as monotherapy in heavily pretreated patients and in combination with standard regimens in earlier treatment lines. In phase-II and III trials, daratumumab elicited an overall response rate (ORR) exceeding 30% as monotherapy and improved the progression-free survival (PFS) and overall survival when added to bortezomib- or lenalidomide-based backbones. Subsequently, isatuximab, a distinct anti-CD38 monoclonal antibody with epitope specificity overlapping but not identical to daratumumab’s, showed comparable clinical activity and tolerability (Leleu et al., 2022; Zabaleta et al., 2024; Wang et al., 2024). Nevertheless, heterogeneity in the study designs, patient demographics, dosing regimens, and concomitant treatment backbones hampers direct comparison between trials and limits our ability to draw definitive conclusions regarding the most effective therapeutic strategies.

Accordingly, the present study undertakes a systematic review and meta-analysis to evaluate the efficacy and safety of CD38 monoclonal antibody therapy in MM. By synthesizing high-quality trial data, this analysis seeks to refine the clinical application of CD38-targeted immunotherapy and highlight areas for future investigation.

## 2 Methods

### 2.1 Search strategy

The literature search adhered to the Preferred Reporting Items for Systematic Reviews and Meta-Analyses (PRISMA) guidelines (Page et al., 2021). We queried PubMed, the Cochrane Library, Web of Science, and Embase from inception until 30 June 2025, without language restrictions. Our strategy combined controlled vocabulary (MeSH in PubMed; Emtree in Embase) with free‐text terms to identify the randomized controlled trials (RCTs) of CD38‐targeted therapies in MM. Key concepts and synonyms included “Cluster of differentiation 38” OR “CD38” OR “daratumumab” OR “isatuximab” OR “anti‐CD38” OR “CD38 monoclonal antibody,” AND “multiple myeloma” OR “plasma cell myeloma,” AND “randomized controlled trial” OR “randomized trial.” We supplemented database searches by screening the reference lists of pertinent publications and reviewing clinical trial registries and conference proceedings to capture any additional eligible studies. Detailed, database‐specific search syntaxes are presented in Supplementary Table S1. This study was registered in PROSPERO (registration number: CRD420251231821) after its completion.

### 2.2 Inclusion and exclusion criteria

The inclusion criteria were as follows: (1) adult patients (≥18 years) with a histologically or cytologically confirmed diagnosis of MM; (2) treatment with a CD38‐targeted monoclonal antibody (e.g., daratumumab or isatuximab) administered as monotherapy or in combination with standard antimyeloma regimens; (3) a comparator arm based on proteasome inhibitor- or immunomodulatory agent-containing therapy; (4) reporting of at least one efficacy endpoint (ORR (Mateos et al., 2020), complete response (Maranduca et al., 2024) rate, PFS, or OS) and safety data (grade ≥3 adverse events); and (5) randomized controlled trial design published in full text through 30 June 2025, without language restriction. The exclusion criteria comprised (1) a non-randomized study designs, including observational cohorts, single‐arm phase-I/II trials, case reports, and case series; (2) preclinical investigations (animal studies, *in vitro* experiments, or pharmacokinetic/pharmacodynamic analyses); (3) trials lacking extractable data for the specified efficacy or safety endpoints; and (4) duplicate reports of overlapping patient cohorts, in which case only the most comprehensive or most recent publication was retained.

### 2.3 Literature screening and data extraction

Data extraction was performed independently by two reviewers in accordance with the predefined inclusion and exclusion criteria. Initially, the titles and abstracts of all retrieved records were screened to exclude clearly ineligible studies; full texts were then obtained for the remaining articles to confirm eligibility. Each reviewer independently extracted the following data using a standardized form: the first author, year of publication, number of prior lines of therapy, median follow-up duration, sample size, patients’ mean or median age, intervention details (type and dosing of CD38 monoclonal antibody and comparator regimen), and pre-specified efficacy and safety outcomes. Extracted data were cross-checked for consistency, and any discrepancies were resolved through discussion; for both full-text screening and data extraction, any disagreements between the two reviewers were resolved through discussion, and if consensus could not be reached, a third reviewer adjudicated the disagreement.

### 2.4 Quality assessment

The risk of bias was assessed independently by two reviewers using the Cochrane risk of bias 2.0 (RoB 2.0) tool for randomized trials. Each study was evaluated across the five RoB 2.0 domains—(1) bias arising from the randomization process, (2) bias due to deviations from the intended interventions, (3) bias due to missing outcome data, (4) bias in the measurement of the outcome, and (5) bias in the selection of the reported result—with judgments of “low risk,” “some concerns,” or “high risk” assigned to each domain (Sterne et al., 2019).

### 2.5 Statistical analyses

Statistical analyses were performed using Review Manager (RevMan) version 5.4 and Stata version 18.0. Between‐study heterogeneity was evaluated by Cochran’s Q test and quantified with the I^2^ statistic; a fixed‐effect model was applied when I^2^ ≤ 50%, and a random‐effects model was used when I^2^ > 50%. Sensitivity analyses were conducted by sequentially omitting individual studies to assess the stability of the pooled estimates. Publication bias was examined using Egger’s regression asymmetry test. All statistical tests were two‐tailed, and a *p*‐value <0.05 was considered indicative of statistical significance.

## 3 Results

### 3.1 Search results and study selection

**FIGURE 1:**
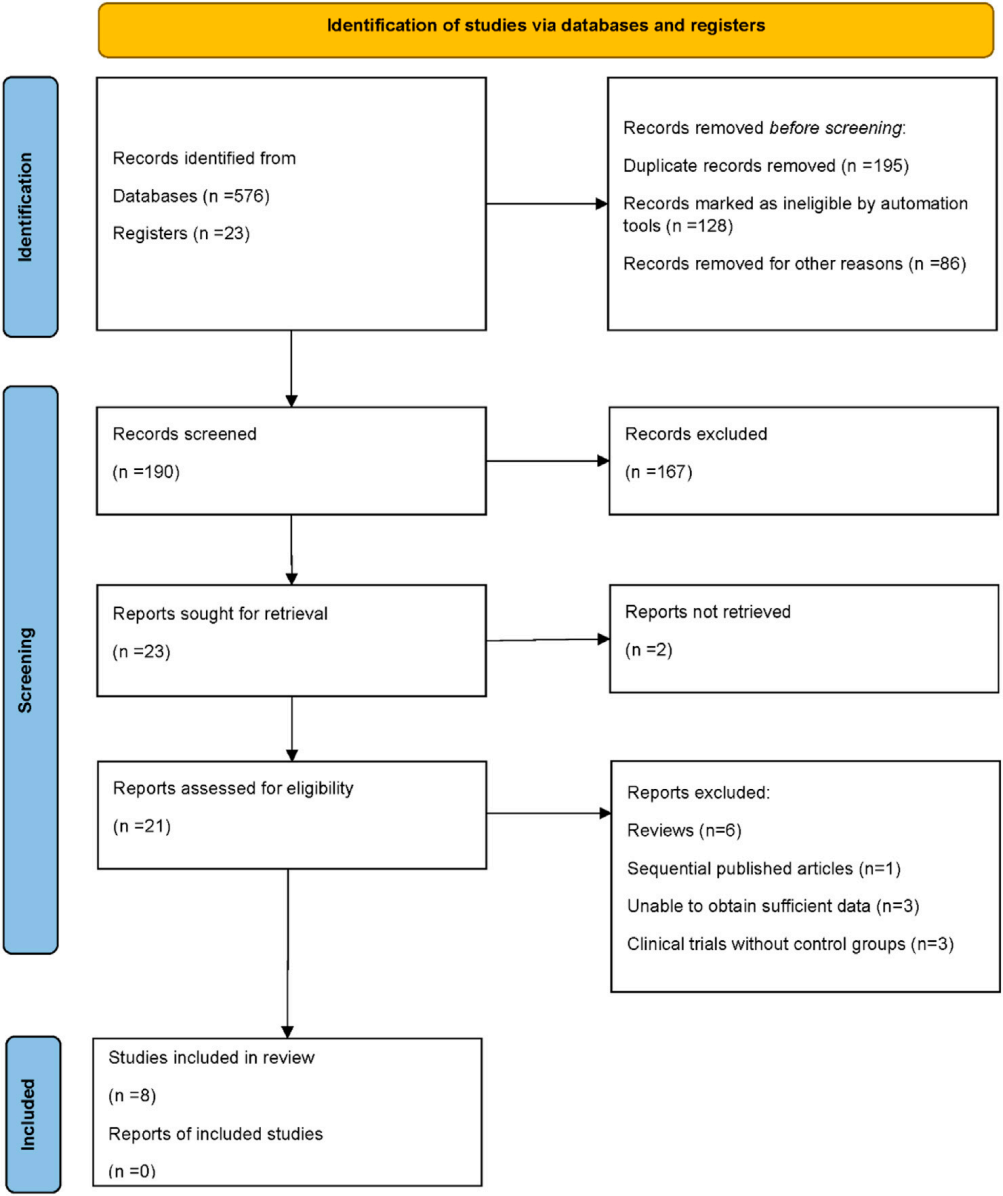
PRISMA flow diagram of study identification, screening, eligibility assessment, and inclusion.

A total of 599 records were retrieved through our comprehensive database and registry searches (576 from electronic databases and 23 from clinical trial registers). Prior to formal screening, 409 records were removed: 195 were duplicates, 128 were marked ineligible by automation tools (which included tools that detected non-randomized studies, irrelevant articles based on keywords, and articles not focused on CD38-targeted therapies), and 86 were excluded for other reasons, including records with insufficient metadata or incomplete references that could not be further assessed. Of the remaining 190 records, 167 were excluded after title and abstract review, leaving 23 articles for full-text assessment. Two reports could not be obtained, and the full texts of the remaining 21 studies were evaluated for eligibility. Thirteen publications were subsequently excluded for the following reasons: six were review articles, one represented a sequential publication from an overlapping cohort, three lacked sufficient data for extraction, and three were single-arm trials without control groups. Ultimately, eight randomized controlled trials met all the inclusion criteria and were included in the quantitative synthesis (Mateos et al., 2020; Martin et al., 2022; Dimopoulos M. et al., 2021; Attal et al., 2019; Dimopoulos et al., 2020; Bahlis et al., 2020; Dimopoulos MA. et al., 2021; Lu et al., 2021) (Figure 1).

### 3.2 Study characteristics

Eight randomized controlled trials published between 2019 and 2022 were included, comprising a total of 2,821 patients with relapsed or refractory MM. Sample sizes ranged from 164 to 569 participants, with the median ages in both the intervention and control arms clustered between 61 and 68 years. Prior lines of therapy varied across studies, ranging from one or more lines in five trials to two or more lines in one trial and three or more lines in one trial; one study did not report this parameter. Follow-up durations spanned 8.2–44.3 months, reflecting both shorter safety assessments and longer efficacy evaluations. Intervention regimens combined a CD38‐targeted antibody (daratumumab or isatuximab) with backbone therapies—pomalidomide, carfilzomib, bortezomib, or lenalidomide plus dexamethasone—while the control groups received the same backbone without the CD38 antibody (Table 1).

**TABLE 1:** Characteristics of the included randomized controlled trials.

First author	Year	Prior lines of therapy	Follow-up (months)	Sample size (n)	Age—Intervention (years, range)	Age—Control (years, range)	Intervention regimen (n)	Control regimen (n)
Dimopoulos M	2021	≥3	—	164	66 (42–85)	68 (37–84)	Id (n = 55)	I (n = 109)
Dimopoulos M. A	2021	≥1	16.9	304	67 (42–86)	68 (35–90)	DPd (n = 151)	Pd (n = 153)
Lu	2021	≥1	8.2	211	61 (28–79)	61 (43–82)	DVd (n = 141)	Vd (n = 70)
Martin	2022	1–3	20.7	302	64 (33–90)	64 (33–90)	IKd (n = 179)	Kd (n = 123)
Dimopoulos	2020	1–3	17	466	64 (57–70)	65 (59–71)	DKd (n = 312)	Kd (n = 154)
Mateos	2020	≥1	40	498	64 (30–88)	64 (33–85)	DVd (n = 251)	Vd (n = 247)
Bahlis	2020	≥1	44.3	569	65 (34–89)	65 (42–87)	DRd (n = 286)	Rd (n = 283)
Attal	2019	≥2	11.6	307	68 (60–74)	66 (59–71)	IPd (n = 154)	Pd (n = 153)

IKd, isatuximab + carfilzomib + dexamethasone; Kd, carfilzomib + dexamethasone; Id, isatuximab + dexamethasone; I, isatuximab; IPd, isatuximab + pomalidomide + dexamethasone; Pd, pomalidomide + dexamethasone; DKd, daratumumab + carfilzomib + dexamethasone; DVd, daratumumab + bortezomib + dexamethasone; Vd, bortezomib + dexamethasone; DRd, daratumumab + lenalidomide + dexamethasone; Rd, lenalidomide + dexamethasone.

### 3.3 Results of quality assessment

**FIGURE 2:**
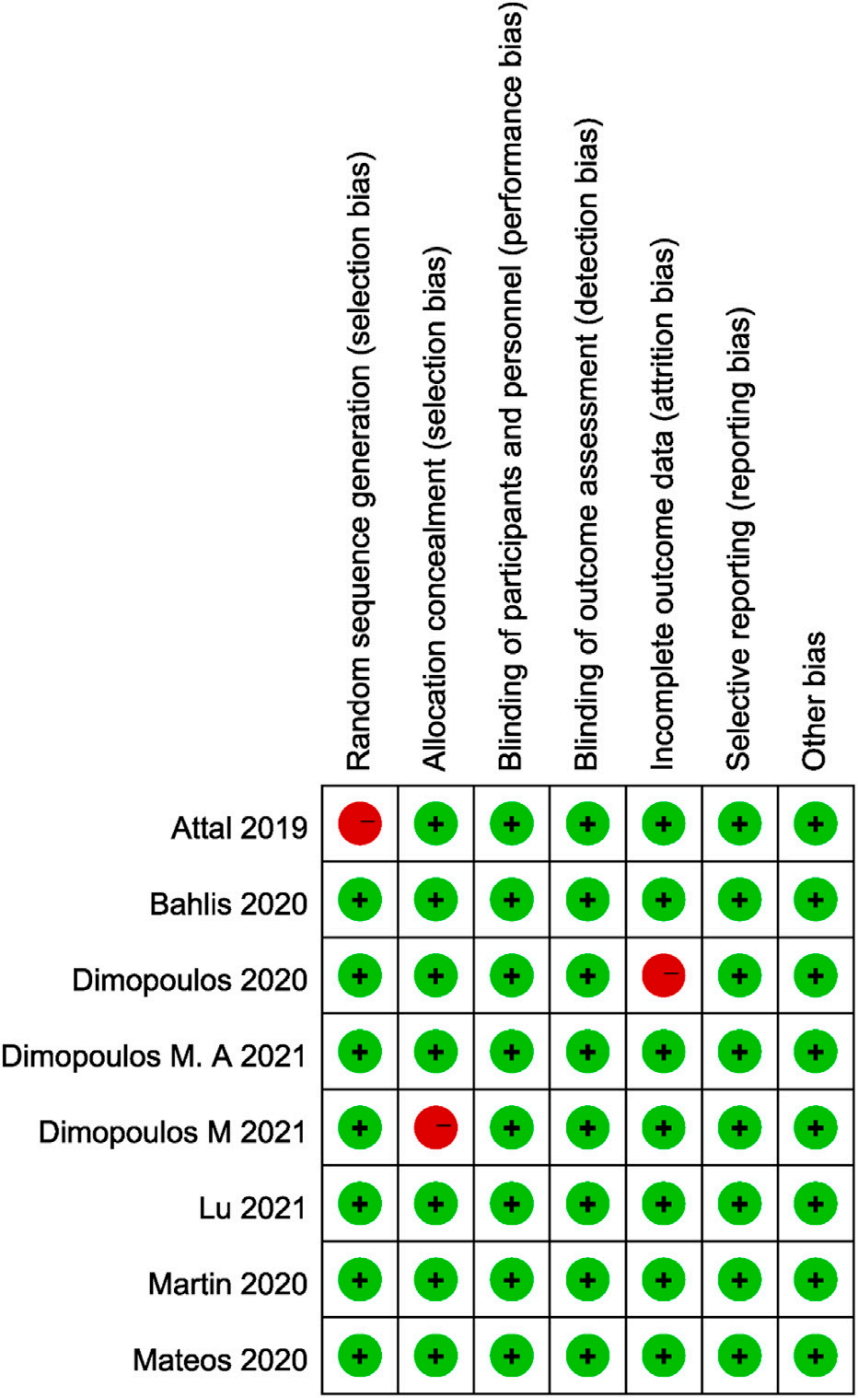
Risk of bias summary for the included trials across the five RoB 2.0 domains.

Across the eight randomized controlled trials, the risk of bias was predominantly low in all five core RoB 2.0 domains. Most studies clearly described random sequence generation and allocation concealment, with only one trial lacking sufficient detail on each of these processes. Blinding of participants, personnel, and outcome assessors was uniformly implemented, thus minimizing performance and detection bias. All trials adequately addressed incomplete outcome data, with only a single study raising minor concerns regarding attrition bias. No evidence of selective reporting or other systematic biases was identified. Overall, the methodological rigor of the included trials was high, supporting confidence in the validity of the meta‐analytic findings (Figure 2).

### 3.4 Overall response rate

**FIGURE 3:**
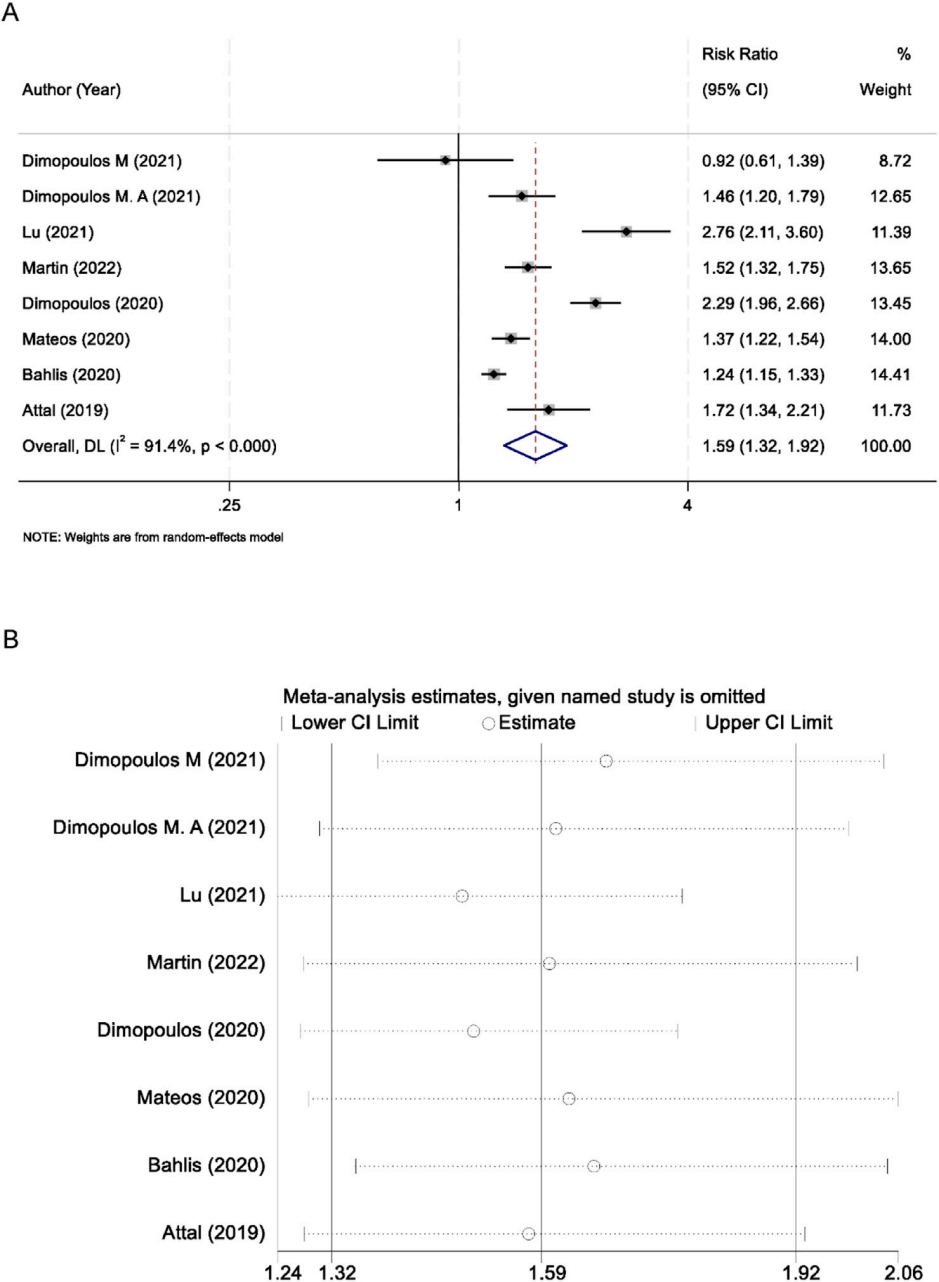
Forest plot of the overall response rate comparing CD38-targeted therapy versus control **(A)**, with inset leave-one-out sensitivity analysis **(B)**.

All eight included trials reported data on ORR. Substantial between‐study heterogeneity was observed (I^2^ = 91.4%; *p* < 0.001), prompting the use of a random‐effects model. The pooled analysis demonstrated that CD38‐targeted therapy significantly improved ORR compared with control regimens (RR 1.59; 95% CI 1.32–1.92; *p* < 0.001) (Figure 3A). To assess the stability of this effect estimate, we conducted leave‐one‐out sensitivity analyses. Sequential exclusion of each trial did not materially alter the pooled RR, indicating that no single study unduly influenced the overall result (Figure 3B).

Subgroup analyses further supported the superiority of CD38‐targeted therapy across clinically relevant patient strata (Table 2). Patients with both impaired (glomerular filtration rate [GFR] ≤ 60 mL min^−1^·1.73 m^−2^) and preserved renal function (GFR >60 mL min^−1^·1.73 m^−2^) derived significant ORR benefit (RRs 1.59 and 1.66, respectively; both *p* < 0.001), despite substantial heterogeneity in the latter subgroup. Age subgroups (<65 and ≥65 years) also demonstrated comparable improvements in response (RRs 1.52 and 1.54; both *p* < 0.001). When stratified by the ISS stage, the magnitude of ORR enhancement was the greatest in stage I of the disease (RR 1.75; *p* < 0.001) and remained significant in stages II and III (RRs 1.68 and 1.42; both *p* < 0.001). Finally, patients receiving one, two, three, or more than three prior lines of therapy all experienced significantly higher response rates with CD38 antibodies (RRs ranging from 1.38 to 1.59; all *p* ≤ 0.006). In addition, a pre-specified subgroup analysis comparing isatuximab‐containing and daratumumab‐containing regimens demonstrated that both antibody types were associated with meaningful improvements in ORR relative to the standard therapy. Isatuximab combinations yielded a pooled RR of 1.48, whereas daratumumab‐based regimens produced a pooled RR of 1.62, with both analyses showing statistically significant benefits (all *p* < 0.001) (Table 2).

**TABLE 2:** Subgroup analyses for the overall response rate.

Subgroup	No. of studies	Heterogeneity (I^2^, *p*)	Model	RR (95% CI)	*p*-value
Renal function					
GFR ≤60 mL min^−1^·1.73 m^−2^	5	0%, 0.75	Fixed effect	1.59 (1.35–1.87)	<0.001
GFR >60 mL min^−1^·1.73 m^−2^	3	77%, 0.002	Random effects	1.66 (1.30–2.12)	<0.001
Age					
<65 years	4	53%, 0.06	Random effects	1.52 (1.25–1.84)	<0.001
≥65 years	4	73%, 0.005	Random effects	1.54 (1.23–1.93)	<0.001
ISS stage					
Stage I	3	70%, 0.01	Random effects	1.75 (1.33–2.30)	<0.001
Stage II	2	11%, 0.34	Fixed effect	1.68 (1.42–1.99)	<0.001
Stage III	3	0%, 0.42	Fixed effect	1.42 (1.20–1.68)	<0.001
Number of prior therapies					
1	1	77%, 0.001	Random effects	1.59 (1.24–2.05)	<0.001
2	2	0%, 0.75	Fixed effect	1.52 (1.25–1.85)	<0.001
3	2	0%, 0.87	Fixed effect	1.45 (1.11–1.90)	0.006
>3	3	0%, 0.44	Fixed effect	1.38 (1.16–1.63)	<0.001
Isatuximab-containing regimens	3	42%, 0.17	Fixed effect	1.48 (1.22–1.80)	<0.001
Daratumumab-containing regimens	5	68%, 0.01	Random effects	1.62 (1.30–2.05)	<0.001

GFR, glomerular filtration rate; ISS, international staging system; RR, risk ratio; CI, confidence interval.

### 3.5 Progression-free survival

**FIGURE 4:**
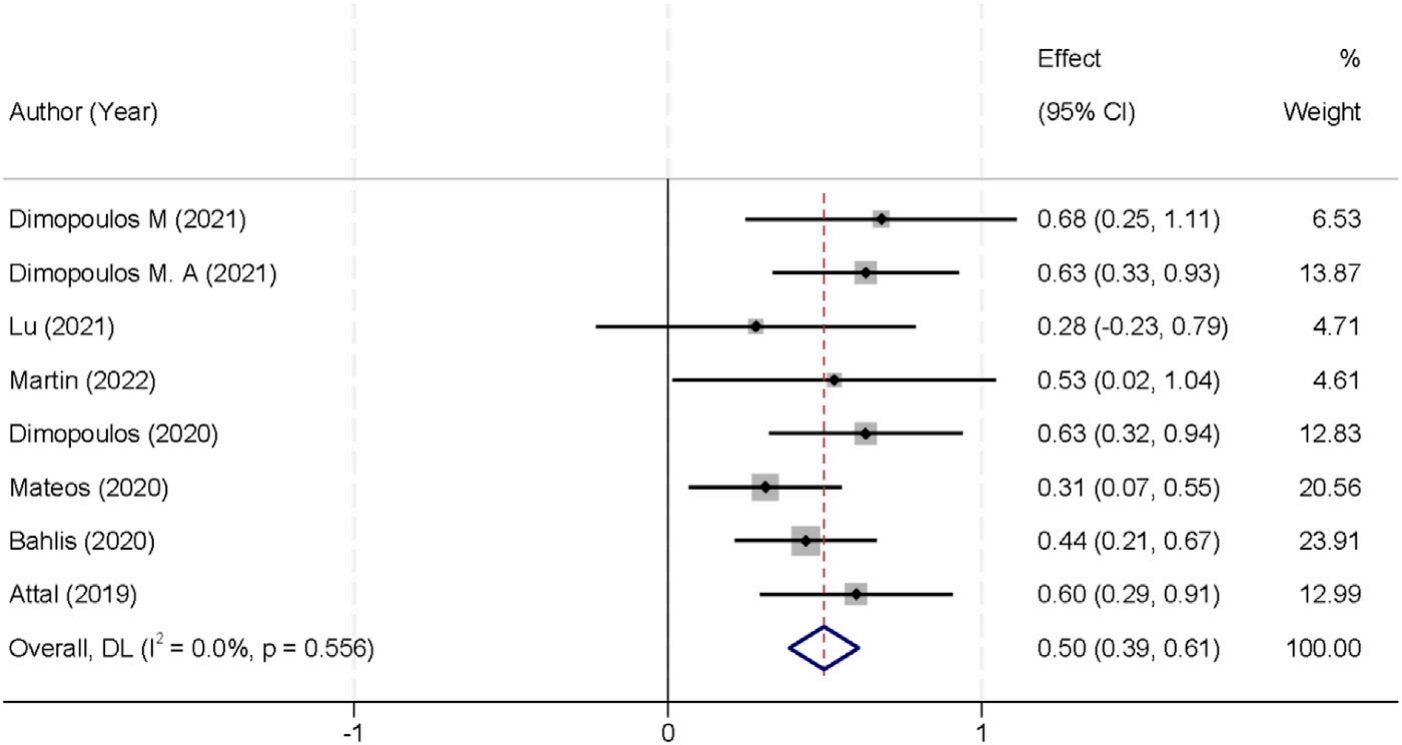
Forest plot of progression-free survival comparing CD38-targeted therapy versus control.

PFS data were available from all eight randomized controlled trials. Statistical heterogeneity was negligible (I^2^ = 0.0%; *p* = 0.556), allowing for a fixed-effect meta-analysis. The pooled HR demonstrated a 50% reduction in the risk of disease progression or death with CD38-targeted therapy compared to that with control regimens (HR 0.50; 95% CI 0.39–0.61; *p* < 0.001) (Figure 4).

### 3.6 Additional response outcomes

Meta‐analysis using a random‐effects model demonstrated that CD38‐targeted therapy significantly increased the rates of very good partial response or better (≥VGPR; RR 1.86; 95% CI 1.53–2.27; *p* < 0.00001; I^2^ = 79%) and complete response or better (≥CR; RR 2.57; 95% CI 1.89–3.50; *p* < 0.001; I^2^ = 63%). Conversely, partial response (PR) was less frequent in the experimental arms (RR 0.67; 95% CI 0.53–0.86; *p* = 0.002; I^2^ = 64%), reflecting deeper remissions. Importantly, CD38 antibodies markedly improved the minimal residual disease (MRD) negativity (RR 5.28; 95% CI 2.80–9.96; *p* < 0.001; I^2^ = 65%) (Table 3).

**TABLE 3:** Meta-analysis of the additional response outcomes.

Endpoint	No. of studies	Experimental rate (n/N, %)	Heterogeneity (I^2^, *p*)	Model	RR (95% CI)	*p*-value
≥VGPR	8	—	79%, <0.0001	Random effects	1.86 (1.53–2.27)	<0.00001
PR	8	267/1,509 (17.7%)	64%, 0.006	Random effects	0.67 (0.53–0.86)	0.002
MRD negative	5	245/1,169 (20.96%)	65%, 0.02	Random effects	5.28 (2.80–9.96)	<0.001
≥CR	7	480/1,454 (33.01%)	63%, 0.01	Random effects	2.57 (1.89–3.50)	<0.001

VGPR, very good partial response; PR, partial response; MRD, minimal residual disease; CR, complete response; RR, risk ratio; CI, confidence interval.

### 3.7 Safety outcomes

All‐grade non‐hematologic adverse events are summarized in Table 4. Compared with control regimens, CD38‐targeted therapy was associated with significantly higher rates of upper respiratory tract infection (RR 1.55; 95% CI 1.36–1.77; *p* < 0.001; I^2^ = 0%), pneumonia (RR 1.34; 95% CI 1.13–1.59; *p* < 0.001; I^2^ = 0%), bronchitis (RR 1.64; 95% CI 1.07–2.51; *p* = 0.021; I^2^ = 54%), diarrhea (RR 1.49; 95% CI 1.33–1.68; *p* < 0.001; I^2^ = 0%), and back pain (RR 1.29; 95% CI 1.07–1.57; *p* = 0.009; I^2^ = 48%). The rates of dyspnea, constipation, hypertension, fatigue, and insomnia did not differ significantly between the arms (all *p* > 0.05).

**TABLE 4:** Incidence of all-grade non-hematologic adverse events.

Adverse event	Studies (n)	Experimental incidence (n/N [%])	Heterogeneity I^2^ (p)	Meta-analysis model	Risk ratio (95% CI)	*p*-value
Upper respiratory tract infection	8	498/1,507 (33.0)	0% (0.78)	Fixed effect	1.55 (1.36–1.77)	<0.001
Pneumonia	8	287/1,507 (19.0)	0% (0.96)	Fixed effect	1.34 (1.13–1.59)	<0.001
Bronchitis	4	137/667 (20.5)	54% (0.09)	Random effects	1.64 (1.07–2.51)	0.021
Dyspnea	4	141/692 (20.4)	10% (0.34)	Fixed effect	1.09 (0.86–1.39)	0.463
Diarrhea	8	543/1,507 (36.0)	0% (0.27)	Fixed effect	1.49 (1.33–1.68)	<0.001
Constipation	5	197/873 (22.6)	55% (0.07)	Random effects	1.00 (0.73–1.36)	0.865
Hypertension	4	210/868 (24.2)	71% (0.02)	Random effects	1.61 (1.00–2.58)	0.052
Back pain	5	197/910 (21.7)	48% (0.10)	Fixed effect	1.29 (1.07–1.57)	0.009
Fatigue	7	364/1,367 (26.6)	16% (0.31)	Fixed effect	1.13 (0.99–1.29)	0.081
Insomnia	4	161/655 (24.6)	77% (0.005)	Random effects	1.64 (0.92–2.92)	0.092

Grade ≥3 non‐hematologic events are presented in Table 5. Severe upper respiratory tract infection (RR 1.99; 95% CI 1.15–3.43; *p* = 0.01; I^2^ = 0%), pneumonia (RR 1.30; 95% CI 1.05–1.62; *p* = 0.02; I^2^ = 0%), diarrhea (RR 2.44; 95% CI 1.58–3.76; *p* < 0.001; I^2^ = 16%), and fatigue (RR 1.75; 95% CI 1.19–2.56; *p* = 0.004; I^2^ = 0%) occurred more often in the experimental arms. Grade ≥3 bronchitis, dyspnea, hypertension, back pain, and insomnia did not show significant differences.

**TABLE 5:** Incidence of grade ≥3 non-hematologic adverse events.

Adverse event	Studies (n)	Experimental incidence (n/N [%])	Heterogeneity I^2^ (*p*)	Model	Risk ratio (95% CI)	*p*-value
Upper respiratory infection	8	50/1,507 (3.3)	0% (0.48)	Fixed effect	1.99 (1.15–3.43)	0.01
Pneumonia	8	194/1,507 (12.9)	0% (0.78)	Fixed effect	1.30 (1.05–1.62)	0.02
Bronchitis	4	19/667 (2.8)	37% (0.19)	Fixed effect	1.61 (0.79–3.27)	0.19
Dyspnea	4	27/692 (3.9)	0% (0.42)	Fixed effect	2.14 (1.00–4.58)	0.05
Diarrhea	8	75/1,507 (5.0)	16% (0.30)	Fixed effect	2.44 (1.58–3.76)	<0.001
Hypertension	4	123/868 (14.1)	69% (0.02)	Random effects	1.87 (0.94–3.74)	0.08
Back pain	5	20/910 (2.2)	0% (0.91)	Fixed effect	1.54 (0.77–3.11)	0.22
Fatigue	7	79/1,367 (5.8)	0% (0.65)	Fixed effect	1.75 (1.19–2.56)	0.004
Insomnia	4	17/655 (2.6)	0% (0.88)	Fixed effect	1.90 (0.84–4.32)	0.13

Hematologic toxicities are detailed in Table 6. Severe thrombocytopenia was more frequent with CD38‐targeted therapy (RR 1.10; 95% CI 1.01–1.20; *p* = 0.02; I^2^ = 50%), while the rates of lymphopenia, anemia, and neutropenia were not significantly different between the groups (all *p* > 0.05).

**TABLE 6:** Incidence of grade ≥3 hematologic adverse events.

Endpoint	No. of studies	Experimental rate (n/N, %)	Heterogeneity I^2^ (*p*)	Model	RR (95% CI)	*p*-value
Lymphopenia	6	228/1,178 (19.4)	73% (<0.10)	Random effects	1.33 (0.96–1.84)	0.08
Anemia	8	826/1,507 (54.8)	51% (0.05)	Random effects	1.00 (0.97–1.03)	0.97
Thrombocytopenia	8	849/1,507 (56.3)	50% (0.05)	Random effects	1.10 (1.01–1.20)	0.02
Neutropenia	8	706/1,507 (46.8)	90% (<0.00001)	Random effects	1.25 (0.99–1.57)	0.06

### 3.8 Publication bias

Egger’s regression analyses demonstrated no evidence of publication bias across all evaluated outcomes, with *p*‐values exceeding 0.05 for each variable tested.

## 4 Discussion

In this systematic review and meta‐analysis of eight randomized controlled trials, CD38‐targeted monoclonal antibody therapy demonstrated substantial improvements in both the response rates and survival outcomes among patients with relapsed or refractory MM. The observed enhancement in ORR (RR 1.59; 95% CI 1.32–1.92; *p* < 0.001) confirms the potent antimyeloma activity of CD38 antibodies when added to standard backbone regimens. The consistency of this benefit across diverse patient subgroups, stratified by renal function, age, ISS stage, and prior treatment exposure, underscores the broad applicability of these agents in routine clinical practice.

CD38-targeted therapies, including daratumumab and isatuximab, demonstrate a significant ORR advantage, driven by mechanisms such as complement-dependent cytotoxicity, antibody-dependent cellular cytotoxicity, and apoptosis induction in CD38-expressing plasma cells. Additionally, CD38 inhibition disrupts adenosine production in the bone marrow, reversing local immune suppression and enhancing T-cell and natural killer (NK) cell activity. Preclinical studies show enhanced myeloma cell killing when combined with proteasome inhibitors or immunomodulatory drugs, which is likely due to synergistic upregulation of CD38 on tumor cells and improved effector recruitment (Perez de Acha et al., 2023; Rosenblatt et al., 2014). Our meta-analysis, including combinations such as daratumumab–bortezomib–dexamethasone and isatuximab–pomalidomide–dexamethasone, confirms these clinical benefits, with pooled ≥VGPR RR 1.86 and ≥CR RR 2.57, which exceed the historical responses from backbone therapies alone. CD38 therapy also significantly prolonged PFS (HR 0.50; 95% CI 0.39–0.61; *p* < 0.001), matching or surpassing the benefits seen with other novel regimens, such as carfilzomib–lenalidomide–dexamethasone (KRd) and pomalidomide–bortezomib–dexamethasone (PVd). Notably, the significant increase in MRD negativity rates (RR 5.28; 95% CI 2.80–9.96) highlights deeper remissions, which are a key predictor of long-term survival. These findings align with subgroup analyses from the POLLUX trial and the CASTOR trial, which reported superior MRD negativity with daratumumab-based regimens, and with clinical cohorts showing that MRD negativity correlates with extended PFS and overall survival (Ebraheem et al., 2024; Horenstein et al., 2025).

The substantial heterogeneity observed in the ORR analysis (I^2^ = 91.4%) can be attributed to several factors. First, differences in the backbone regimens used across the studies, specifically proteasome inhibitors (PIs) versus immunomodulatory drugs (IMiDs), may affect the efficacy of CD38-targeted therapies. PI-based regimens, such as carfilzomib or bortezomib, induce deeper responses, potentially enhancing the benefits of CD38 antibodies, while IMiDs, which modulate immune responses, might interact differently with CD38 therapies, thus influencing ORR. Additionally, the variability in prior therapy lines is another key contributor. Some studies included patients with early relapsed disease, while others focused on heavily pretreated populations. This could explain the observed differences in ORR, with patients receiving fewer prior treatments potentially showing greater benefit from CD38-targeted therapies. The inclusion of both daratumumab and isatuximab, each with distinct pharmacodynamics, dosing regimens, and mechanisms of action, further contributes to this heterogeneity. These subtle differences in treatment protocols may influence the response magnitudes, underscoring the need for careful interpretation of ORR results. Despite this variability, our subgroup analyses addressed important clinical concerns. Patients with moderate-to-severe renal impairment (GFR ≤60 mL min^−1^·1.73 m^2^) derived similar ORR benefits (RR 1.59) to those with preserved renal function (RR 1.66), supporting the efficacy of CD38 antibodies in patients with compromised kidney function. This aligns with pharmacokinetic studies indicating the minimal renal clearance of monoclonal antibodies, suggesting that renal function does not significantly impact the pharmacokinetics or therapeutic efficacy of CD38 antibodies in MM. Subgroup analysis by age (<65 vs. ≥65 years) also showed equivalent response improvements, reinforcing the utility of CD38 therapies in older patients, who often have higher comorbidity burdens. Furthermore, CD38 antibodies demonstrated efficacy across all ISS stages, with the most substantial relative benefit observed in stage I of the disease, which is likely due to more robust immune competence and lower tumor burden in the earlier stages (Shang et al., 2024; Ramasamy et al., 2025). Finally, clinical benefits persisted across all lines of therapy, from the first relapse to heavily pretreated patients (i.e., >3 prior therapies), confirming the efficacy of CD38 antibodies as a backbone therapy in both early and later treatment settings.

CD38 antibodies were generally well-tolerated, with increases in low-grade, non-hematologic adverse events, such as upper respiratory tract infections (RR 1.55) and diarrhea (RR 1.49). Grade ≥3 infections and diarrhea were more frequent (e.g., severe diarrhea RR 2.44), requiring proactive monitoring and early management. CD38 depletion’s immunosuppressive effects on lymphoid subsets likely contribute to the infectious risk; however, treatment discontinuation rates remained low, indicating manageable toxicity. Hematologic events were similar between arms, except for thrombocytopenia (RR 1.10), which is consistent with on-target effects. The safety profile aligns with previous reports, supporting favorable risk–benefit considerations given the significant efficacy gains. Our findings extend prior narrative reviews and smaller meta-analyses by offering comprehensive quantitative estimates for efficacy and safety endpoints, including PFS and MRD negativity. A 2019 pooled analysis of early-phase daratumumab studies reported ORR improvements but did not assess the PFS or response depth. More recent meta-analyses on daratumumab–lenalidomide combinations confirmed ORR and PFS improvements but lacked subgroup detail and did not include emerging agents such as isatuximab (Wang et al., 2021; Ban et al., 2018). By including eight rigorous RCTs with pre-specified subgroup and sensitivity analyses, our study provides a more definitive, nuanced evaluation of CD38 antibody therapy across various clinical settings.

Similar to our findings, the study by Usmani et al. (2025) underscores the potent role of CD38-targeted monoclonal antibodies in improving treatment efficacy. Both studies demonstrate substantial improvements in the response depth and PFS with CD38-targeted therapies. However, our meta-analysis provides a broader context by including a variety of patient populations and therapeutic regimens, further solidifying the robustness of CD38 monoclonal antibody efficacy across different clinical settings. Our results are consistent with those of Voorhees et al. (2020), with both studies showing enhanced response rates and PFS with daratumumab-based regimens. However, our meta-analysis includes a more diverse range of therapeutic contexts, particularly relapsed/refractory patients, thereby providing stronger evidence for the role of CD38 monoclonal antibodies as key components of combination therapy, regardless of the patients’ status or prior treatment history. These comparisons collectively reinforce the growing body of evidence supporting the integration of CD38-targeted monoclonal antibodies as a cornerstone of combination therapy in MM, offering consistent benefits across diverse clinical contexts.

This meta‐analysis provides strong evidence that CD38 monoclonal antibody-based regimens significantly improve the response rates, deepen remissions (including MRD negativity), and prolong PFS in relapsed or refractory MM, with a manageable safety profile. These data support the incorporation of CD38 antibodies as standard components of treatment algorithms across various patient subgroups, regardless of renal function, age, disease stage, or prior therapy burden. Future research should focus on optimizing sequencing strategies, integrating CD38 antibodies into front‐line regimens, and investigating combination approaches that further harness immune effector mechanisms. Several limitations merit consideration. First, the high heterogeneity in ORR and secondary response endpoints (I^2^ up to 79%) reflects variability in the trial designs, patient populations, and concomitant regimens; although random‐effects models were applied, residual confounding cannot be excluded. Second, overall survival data were immature or unavailable in several trials, precluding robust meta‐analysis of this key outcome. Third, MRD assays varied in sensitivity and methodology across studies, potentially influencing the pooled estimates. Finally, while publication bias was not detected by Egger’s test, the small number of included trials for certain endpoints limits the power of such assessments.

## 5 Conclusions

In this meta‐analysis of eight randomized trials, CD38‐targeted monoclonal antibodies significantly improved the overall and deep response rates (VGPR, CR, and MRD negativity) and halved the progression‐free survival risk compared to that of standard regimens across diverse patient subgroups. While non‐hematologic infections and diarrhea increased, severe toxicities remained manageable. These findings support the broad efficacy and acceptable safety of CD38 antibodies in the treatment of relapsed/refractory MM.

## Data Availability

The raw data supporting the conclusions of this article will be made available by the authors, without undue reservation.
