# Supplementary material for: Cluster of differentiation 38 monoclonal antibody therapy in the treatment of multiple myeloma: a systematic review and meta-analysis
Source: Front Pharmacol. 2026 Feb 12;16:1687718. doi: 10.3389/fphar.2025.1687718 (PMC12936039; doi:10.3389/fphar.2025.1687718)
Supplement: Supplementary file 1 [file Table1.docx]

Supplementary Table 1 Search Strategy for Four Databases.

| Database | Search Strategy |
| --- | --- |
| PubMed | (“Cluster of Differentiation 38”[MeSH] OR “CD38”[tiab] OR “daratumumab”[tiab] OR “isatuximab”[tiab] OR “anti-CD38”[tiab] OR “CD38 monoclonal antibody”[tiab]) AND (“Multiple Myeloma”[MeSH] OR “multiple myeloma”[tiab] OR “plasma cell myeloma”[tiab]) AND (“Randomized Controlled Trial”[pt] OR “randomized controlled trial”[tiab] OR “randomised trial”[tiab] OR “RCT”[tiab]) |
| Embase | (‘cd38’/exp OR ‘cluster of differentiation 38’:ti,ab OR ‘cd38’:ti,ab OR ‘daratumumab’:ti,ab OR ‘isatuximab’:ti,ab OR ‘anti-cd38’:ti,ab OR ‘cd38 monoclonal antibody’:ti,ab) AND (‘multiple myeloma’/exp OR ‘multiple myeloma’:ti,ab OR ‘plasma cell myeloma’:ti,ab) AND (‘randomized controlled trial’/exp OR ‘randomized controlled trial’:ti,ab OR ‘randomised trial’:ti,ab OR ‘rct’:ti,ab) |
| Web of Science | TS=(“Cluster of Differentiation 38” OR CD38 OR daratumumab OR isatuximab OR “anti-CD38” OR “CD38 monoclonal antibody”) AND TS=(“multiple myeloma” OR “plasma cell myeloma”) AND TS=(“randomized controlled trial” OR “randomised trial” OR RCT) |
| Cochrane Library | [mh “Cluster of Differentiation 38”] OR CD38:ti,ab,kw OR daratumumab:ti,ab,kw OR isatuximab:ti,ab,kw OR anti-CD38:ti,ab,kw OR “CD38 monoclonal antibody”:ti,ab,kw AND [mh “Multiple Myeloma”] OR “multiple myeloma”:ti,ab,kw OR “plasma cell myeloma”:ti,ab,kw AND (randomized:ti,ab,kw OR randomised:ti,ab,kw OR “controlled trial”:ti,ab,kw OR RCT:ti,ab,kw) |
